# Precision measurement of the Newtonian gravitational constant

**DOI:** 10.1093/nsr/nwaa165

**Published:** 2020-07-22

**Authors:** Chao Xue, Jian-Ping Liu, Qing Li, Jun-Fei Wu, Shan-Qing Yang, Qi Liu, Cheng-Gang Shao, Liang-Cheng Tu, Zhong-Kun Hu, Jun Luo

**Affiliations:** TianQin Research Center for Gravitational Physics & School of Physics and Astronomy, Sun Yat-sen University (Zhuhai Campus), Zhuhai 519082, China; TianQin Research Center for Gravitational Physics & School of Physics and Astronomy, Sun Yat-sen University (Zhuhai Campus), Zhuhai 519082, China; MOE Key Laboratory of Fundamental Physical Quantities Measurement & Hubei Key Laboratory of Gravitation and Quantum Physics, PGMF and School of Physics, Huazhong University of Science and Technology, Wuhan 430074, China; MOE Key Laboratory of Fundamental Physical Quantities Measurement & Hubei Key Laboratory of Gravitation and Quantum Physics, PGMF and School of Physics, Huazhong University of Science and Technology, Wuhan 430074, China; TianQin Research Center for Gravitational Physics & School of Physics and Astronomy, Sun Yat-sen University (Zhuhai Campus), Zhuhai 519082, China; TianQin Research Center for Gravitational Physics & School of Physics and Astronomy, Sun Yat-sen University (Zhuhai Campus), Zhuhai 519082, China; MOE Key Laboratory of Fundamental Physical Quantities Measurement & Hubei Key Laboratory of Gravitation and Quantum Physics, PGMF and School of Physics, Huazhong University of Science and Technology, Wuhan 430074, China; TianQin Research Center for Gravitational Physics & School of Physics and Astronomy, Sun Yat-sen University (Zhuhai Campus), Zhuhai 519082, China; MOE Key Laboratory of Fundamental Physical Quantities Measurement & Hubei Key Laboratory of Gravitation and Quantum Physics, PGMF and School of Physics, Huazhong University of Science and Technology, Wuhan 430074, China; MOE Key Laboratory of Fundamental Physical Quantities Measurement & Hubei Key Laboratory of Gravitation and Quantum Physics, PGMF and School of Physics, Huazhong University of Science and Technology, Wuhan 430074, China; TianQin Research Center for Gravitational Physics & School of Physics and Astronomy, Sun Yat-sen University (Zhuhai Campus), Zhuhai 519082, China

**Keywords:** precision measurement, Newtonian gravitational constant, CODATA

## Abstract

The Newtonian gravitational constant *G*, which is one of the most important fundamental physical constants in nature, plays a significant role in the fields of theoretical physics, geophysics, astrophysics and astronomy. Although *G* was the first physical constant to be introduced in the history of science, it is considered to be one of the most difficult to measure accurately so far. Over the past two decades, eleven precision measurements of the gravitational constant have been performed, and the latest recommended value for *G* published by the Committee on Data for Science and Technology (CODATA) is (6.674 08 ± 0.000 31) × 10^−11^ m^3^ kg^−1^ s^−2^ with a relative uncertainty of 47 parts per million. This uncertainty is the smallest compared with previous CODATA recommended values of *G*; however, it remains a relatively large uncertainty among other fundamental physical constants. In this paper we briefly review the history of the *G* measurement, and introduce eleven values of *G* adopted in CODATA 2014 after 2000 and our latest two values published in 2018 using two independent methods.

## INTRODUCTION

Newton’s law of universal gravitation from *Philosophiae Naturalis Principia Mathematica* [1] in its modern form is known as

(1) \begin{equation*} F=G\frac{Mm}{r^{2}}. \end{equation*}

This equation describes the attractive force between the two masses *M* and *m* separated by the distance *r*. The strength of this force is defined by the constant of proportionality *G*, which is called the gravitational constant. As is well known, *G* is one of the earliest fundamental constants introduced by human beings, and it plays a significant role in the fields of theoretical physics, geophysics, astrophysics and astronomy [2]. However, the measurement precision of the gravitational constant has been improved by only about two orders of magnitude in the past two centuries. To date, more than 200 experiments have been performed to precisely determine the value of *G* [3], with the latest value recommended by the Committee on Data for Science and Technology (CODATA), named CODATA 2014, having a relative standard uncertainty of 4.7 × 10^−5^ in 2016 [4]. In Table 1 we present a highly abbreviated list of relative standard uncertainties *u*_*r*_ of the physical and chemical fundamental constants most commonly used based on the 2010 [5] and 2014 [4] adjustments, respectively. Compared with the CODATA 2010 recommended value, the relative standard uncertainty of the gravitational constant is improved by more than a factor of 2 in the CODATA 2014 adjustment. However, it remains a relatively large uncertainty compared to other fundamental constants.

**Table 1.** The relative standard uncertainties *u*_*r*_ of the recommended values of physical and chemical fundamental constants most commonly used based on the CODATA 2010 and 2014 adjustments. For the latest recommended values, see [4,5].

Quantity	Symbol	2010 *u*_*r*_	2014 *u*_*r*_
Speed of light in a vacuum	*c*, *c*_0_	Exact	Exact
Magnetic constant	μ_0_	Exact	Exact
Electric constant	ε_0_	Exact	Exact
Rydberg constant	*R*_∞_	5.0 × 10^−12^	5.9 × 10^−12^
Conductance quantum	*G*_0_	3.2 × 10^−10^	2.3 × 10^−10^
Fine-structure constant	α	3.2 × 10^−10^	2.3 × 10^−10^
Elementary charge	*e*	2.2 × 10^−8^	6.1 × 10^−9^
Magnetic flux quantum	Φ_0_	2.2 × 10^−8^	6.1 × 10^−9^
Faraday constant	*F*	2.2 × 10^−8^	6.2 × 10^−9^
Planck constant	*h*	4.4 × 10^−8^	1.2 × 10^−8^
Electron mass	*m*_*e*_	4.4 × 10^−8^	1.2 × 10^−8^
Proton mass	*m*_*p*_	4.4 × 10^−8^	1.2 × 10^−8^
Avogadro constant	*N*_*A*_	4.4 × 10^−8^	1.2 × 10^−8^
Molar gas constant	*R*	9.1 × 10^−7^	5.7 × 10^−7^
Boltzmann constant	*k*	9.1 × 10^−7^	5.7 × 10^−7^
Newtonian constant of gravitation	*G*	1.2 × 10^−4^	4.7 × 10^−5^

We give some primary reasons why measuring the gravitational constant is so difficult.

- The gravitational interaction between two objects is extremely weak. Hence, the gravitational signal can be easily overwhelmed by other interfering signals, such as the electromagnetic force, ground vibration, temperature fluctuation and so on. Scientists need to spend much effort to design and operate the experimental device so that it overcomes such influences in the *G* measurement.
- The gravitational interaction cannot be shielded. This kind of phenomenon makes a gravitationally precise measurement difficult to decouple from the environmental influences, such as human activity, groundwater, mountains, buildings and other objects.
- To date, there is no quantitatively theoretical relationship between the Newtonian gravitational constant and other fundamental constants. Scientists can only measure the gravitational constant through Newton’s law of universal gravitation. One of the greatest difficulties in any *G* measurement is determining with sufficient accuracy of the dimensions and density distribution of the test mass and attractor mass.

## HISTORY OF THE *G* MEASUREMENT

**Figure 1.**
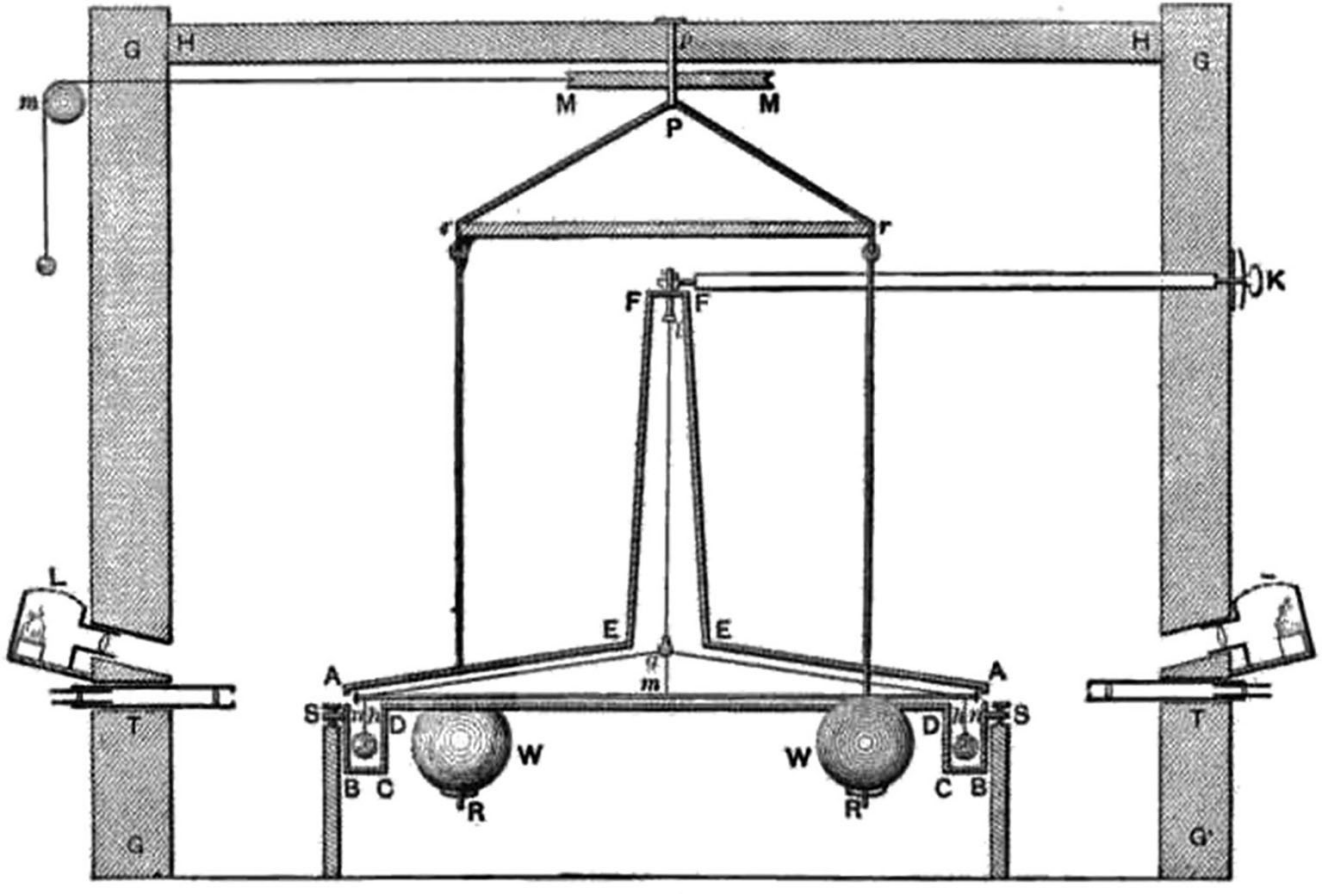
Cavendish’s instrument in the first laboratory measurement of *G*. Adapted from [7].

Hundreds of years after Newton’s discovery of the law of universal gravitation, Henry Cavendish, an outstanding scientist at Cambridge University, performed the first experimental measurement in 1797-98 of the force of gravity between masses in the laboratory [6]. The apparatus, shown in Fig. 1, was designed and built by Rev. John Michell, the purpose of which at the beginning was to determine the density of the Earth. Unfortunately, he did not complete or carry out any experiments before he died in 1793. The device was then forwarded to Cavendish [7,8]. The instrument consists of a torsion balance and two larger spheres as source masses. The torsion balance is made of a rod suspended from a fibre with two smaller spheres as the test masses attached to each end. The source masses are located near the smaller spheres and hung from a separate suspension system. Their mutual attraction caused by the gravitational torque rotates the torsion balance, and the rod stops moving when it reaches a deflection angle where the twisting force of the fibre equals the gravitational force between two kinds of masses. The twist angle of the rod can be measured by calculating the twisting force of the fibre. Hence, it is possible to determine the force between the test masses and the source masses. One of the first references to *G* was made in 1873 [9]. After converting to the international system of units, Cavendish’s value for the density of the Earth is (5.448 ± 0.033) g cm^−3^, yielding *G* = (6.67 ± 0.07) × 10^−11^ m^3^ kg^−1^  s^−2^ with a relative uncertainty of 10^4^ parts per million (ppm).

Cavendish’s experiment is one of the classical experiments in the history of physics. He carried out creative work in this area. The important contribution was to magnify the weak gravitational interaction through ingenious experimental design, and to transform it into an observable signal as the twist angle of the torsion balance. The measurement accuracy has not been improved in the following 100 years. Under the technological conditions of the eighteenth century such experimental precision is a great achievement.

After Cavendish’s experiment, many methods were used to measure the gravitational constant. In order to generate the obvious gravitational signal, experimenters usually used mountains, lakes and other natural massive objects as the source masses to measure *G* values at the early stage [10,11]. However, the relative uncertainty of these kinds of methods was only at a level of 10^3^ ppm because of the difficulty to precisely estimate the mass distribution of natural objects. Consequently, precise measurements of the gravitational constant, using well-designed and well-characterized masses, were mainly performed in the laboratory.

At the beginning of the twentieth century, the accepted *G* value was 6.66 × 10^−11^ m^3^ kg^−1^ s^−2^, which depended on the experiments of Boys [12] and Braun [13]. CODATA, which concentrates on providing a set of recommended fundamental constants, was established in 1966. All the recommended values of the gravitational constant [4,5,14–18] are listed in Table 2.

**Table 2.** All of the CODATA recommended values of *G*.

CODATA	*G* (× 10^−11^ m^3^ kg^−1^ s^−2^)	*u*_*r*_ (ppm)
CODATA 1973 [14]	6.6720 ± 0.0041	615
CODATA 1986 [15]	6.672 59 ± 0.000 85	128
CODATA 1998 [16]	6.673 ± 0.010	1500
CODATA 2002 [17]	6.6742 ± 0.0010	150
CODATA 2006 [18]	6.674 28 ± 0.000 67	100
CODATA 2010 [5]	6.673 84 ± 0.000 80	120
CODATA 2014 [4]	6.674 08 ± 0.000 31	47

The first CODATA recommended value of *G*, named CODATA 1973 [14], is (6.6720 ± 0.0041) × 10^−11^ m^3^ kg^−1^ s^−2^ with a relative uncertainty of 615 ppm, which was based on the result of Heyl’s measurement in 1930 [19] and Heyl and Chrzanowski’s measurement in 1942 [20], with a to-rsion balance using the time-of-swing method. This value is more than one order of magnitude lower than Cavendish’s value. In 1982, at the National Institute of Standards and Technology, Luther and Towler obtained the value *G* = (6.672 59 ± 0.000 43) × 10^−11^ m^3^ kg^−1^ s^−2^ with a relative uncertainty of 64 ppm [21] using the same method. The CODATA Task Group then adopted this value as the CODATA 1986 recommended value [15], *G* = (6.672 59 ± 0.000 85) × 10^−11^ m^3^ kg^−1^ s^−2^, but with its uncertainty doubled to 128 ppm.

In the following decade, nine independent experiments for measuring the value of *G* were performed [22–30]. Unfortunately, they did not agree well with each other and also differed from the CODATA 1986 recommended value. It is suggested that a latest value for *G* should be recommended. What is unexpected, however, is that an anomalous CODATA adjustment occurred in 1998. The CODATA 1998 recommended value of *G* is (6.673 ± 0.010) × 10^−11^ m^3^ kg^−1 ^s^−2^ [16]. It is noted that this value is essentially the same as the CODATA 1986 value, but the relative uncertainty is accidentally increased from 128 ppm to 1500 ppm, which is about a factor of 12 larger. After realizing the seriousness of measuring *G*, scientists made much more effort finding and solving systematic errors when improving previous experimental methods. Several precise values of *G* were published between 2000 and 2010 [31–39]. According to the addition of these values of *G*, the CODATA 2002, CODATA 2006 and CODATA 2010 recommended values are (6.6742 ± 0.0010) × 10^−11^ m^3^ kg^−1 ^s^−2^ [17], (6.674 28 ± 0.000 67) × 10^−11^ m^3^ kg^−1 ^s^−2^ [18] and (6.673 84 ± 0.000 80) × 10^−11^ m^3^ kg^−1 ^s^−2^ [5], respectively. It is worth noting that the uncertainties have significantly improved over the CODATA 1998 recommended value; however, they are still kept at a level of 100 ppm without remarkable progress. This kind of situation could be mainly attributed to the large discrepancy among all of the experimental data from different groups. In 2014, three latest values of *G* obtained by different methods were published, named BIPM-14 [40,41], LENS-14 [42,43] and UCI-14 [44]. These new results did not resolve the considerable disagreements that have existed among the measurements of *G* for the past 20 years. The weighted mean of the fourteen values of *G* [21,25,26,31–39,41,42,44] are adopted in the CODATA 2014 recommended value, which is (6.674 08 ± 0.000 31) × 10^−11^ m^3^ kg^−1 ^s^−2^ with a relative uncertainty of 47 ppm [4]. Compared with the uncertainties of previous recommended values, it is improved by a factor of 2. But this value remains the least precisely known among all of the fundamental constants. Meanwhile, because of the three new values of *G*, the CODATA 2014 recommended value is larger than the CODATA 2010 value by 36 ppm.

**Figure 2.**
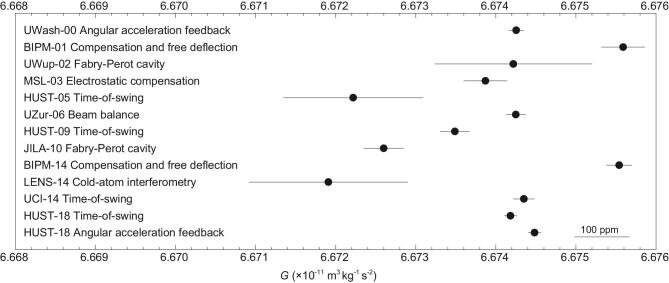
The eleven values of *G* adopted in CODATA 2014 after 2000 and our latest two values published in 2018.

The phenomenon of inconsistent measurements of *G* makes many scientists puzzled [45–49]. It is most likely that there might be some undiscovered systematic errors in some or all the *G* measurements. Our group has been dedicated to the precise measurement of *G* for over thirty years. In 2018, *G* values measured with two independent methods, the time-of-swing (ToS) method and angular acceleration feedback (AAF) method, were obtained with the smallest uncertainty reported to date and both agreed with the CODATA 2014 recommended value to within two standard deviations [50]. The thirteen values of the Newtonian gravitational constant [30–44,50,51] measured after 2000 are listed in Table 3 and shown in Fig. 2. Further experimental details are given in the next section.

**Table 3.** The thirteen values of *G* measured after 2000.

No.	Identification	Method	*G* (× 10^−11^ m^3^ kg^−1 ^s^−2^)	*u*_*r*_ (ppm)
1	UWash-00 [31]	Angular acceleration feedback	6.674 255(92)	14
2	BIPM-01 [32]	Electrostatic compensation and free deflection	6.675 59(27)	40
3	UWup-02 [33]	Fabry–Pérot cavity	6.674 22(98)	150
4	MSL-03 [34]	Electrostatic compensation	6.673 87(27)	40
5	HUST-05 [30,35]	Time of swing	6.672 22(87)	130
6	UZur-06 [36]	Beam balance	6.674 25(12)	19
7	HUST-09 [37,38]	Time of swing	6.673 49(18)	27
8	JILA-10^1^ [39,51]	Fabry–Pérot cavity	6.672 60(25)	37
9	BIPM-14 [40,41]	Electrostatic compensation and free deflection	6.675 54(16)	24
10	LENS-14 [42,43]	Cold-atom interferometry	6.671 91(99)	150
11	UCI-14 [44]	Time of swing	6.674 35(13)	19
12	HUST-18 [50]	Time of swing	6.674 184(78)	12
13	HUST-18 [50]	Angular acceleration feedback	6.674 484(78)	12

^1^JILA-10 value of *G* published in 2010, was corrected to the latest value due to the two calculation errors identified in 2019.

## REVIEW OF MODERN EXPERIMENTS AFTER 2000

Many reviews are of interest in pursuing the measurement of *G* [3,8,9,52–60]. In this paper, we mainly focus on the values of *G* adopted in CODATA 2014 after 2000 and our latest two values published in 2018 using two independent methods [50].

### UWash-00

The angular acceleration feedback method was first adopted by Rose *et al.* [61] at the University of Virginia in 1969, and they obtained the value of *G* = (6.674 ± 0.012) × 10^−11^ m^3^ kg^−1 ^s^−2^ with a relative uncertainty of 1800 ppm. In 2000, Gundlach and Merkowitz from the University of Washington in

Seattle made some remarkable improvements to overcome the systematic errors in previous measurements [31,62,63] and published the *G* value with a relative uncertainty of only 14 ppm.

**Figure 3.**
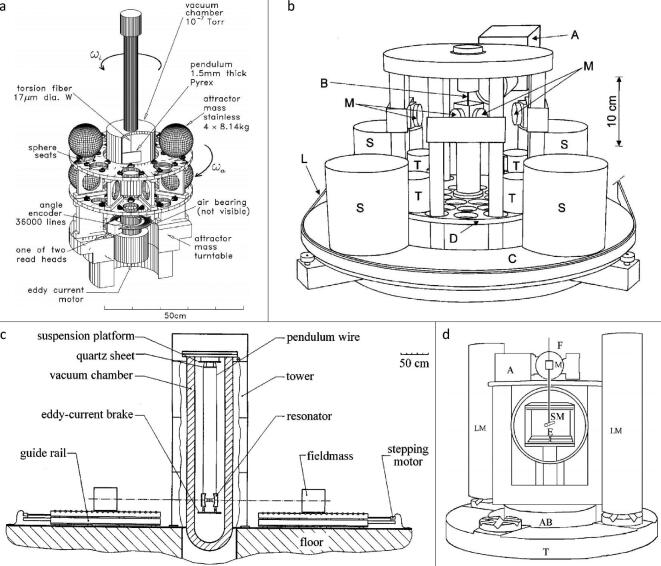
(a) Schematic of the UWash-00 apparatus [31]. Copyright 2000, The American Physical Society. (b) Schematic of the BIPM-01 apparatus [32]. Copyright 2001, The American Physical Society. (c) Schematic of the UWup-02 apparatus [66]. Copyright 1999, IOP Publishing Ltd. (d) Schematic of the MSL-03 apparatus [34]. Copyright 2003, The American Physical Society.

A schematic of the apparatus that Gundlach and Merkowitz used is given in Fig. 3(a). The torsion balance was suspended in the vacuum chamber that was placed on an inner turntable. Two pairs of attractor spheres were located on an outer, separate but coaxial turntable. The inner turntable was first rotated so that the torsion balance experienced a gravitational torque due to the interaction with the source masses. In order to minimize the fibre twist so that the pendulum was stationary relative to the inner turntable, the experimenter turned on the feedback control loop to adjust the rotation rate of the turntable. Finally, the gravitational angular acceleration of the pendulum was made equal to the angular acceleration of the turntable, which could be calculated from the second time derivative of the measured turntable angle. After combining the *G* values obtained with different sets of spheres, the result for the gravitational constant was (6.674 215 ± 0.000 092) × 10^−11^ m^3^ kg^−1 ^s^−2^ with a relative uncertainty of 14 ppm. Subsequently, in 2002, the authors identified an additional fractional correction of 6 ppm due to the magnetic damper [17]. In order to suppress the unwanted mode of the pendulum, the magnetic damper was often used in the torsion balance system. During this method, the pendulum was stationary in the rotating frame due to the feedback loop, but the prehanger fibre that suspended the magnetic damper was still twisted. This effect introduced extra torque to the gravitational torque. Therefore, the value of *G* was corrected to (6.674 255 ± 0.000 092) × 10^−11^ m^3^ kg^−1 ^s^−2^.

The angular acceleration feedback method has three major advantages. First, since the torsion fibre does not experience any appreciable deflection, this method is independent of many torsion fibre properties, especially anelasticity [64]. Second, the source masses are supported by the outer turntable, and the angular velocity is controlled to ensure that the difference in the angular velocity between the inner and outer turntables remains constant. In this way, the gravitational signal generated by the source masses will occur at the difference frequency. Hence, the gravitational interaction due to the environment can be cleanly removed. Third, by choosing the thin flat plate as the pendulum, almost like the two-dimensional plate, the angular acceleration signal is independent of its mass distribution. However, comparing with the other methods that we introduce in the following parts, the angular acceleration feedback experimental system is much more complex. The high-precision and high-stability turntables and feedback control loop are the key components. The stability of the angular velocity of the turntables directly affects the accuracy of the measurement results.

### BIPM-01 and BIPM-14

Quinn *et al.* from the International Bureau of Weights and Measures (Bureau International des Poids et Mesures, BIPM) reported two values of *G*, one in 2001 [32] named BIPM-01, and one in 2013 [40,41] named BIPM-14. The two BIPM experiments used the same principle of a flexure strip torsion balance operating in two different modes, the electrostatic compensation mode and free deflection mode (i.e. the Cavendish method).

In the BIPM-01 experiment (see Fig. 3(b)), a four-mass configuration was used in both the test mass system and the source mass system. The test mass was suspended from a torsion strip in order to give much improved stability with practical independence of the material properties. In the compensation mode, the gravitational torque of the source masses was made equal to an electrostatic torque on the test masses. In the deflection mode, the torsion balance experiences the deflection angle that was related to the gravitational torque. The final results were (6.675 53 ± 0.000 40) × 10^−11^ m^3^ kg^−1 ^s^−2^ with a relative uncertainty of 60 ppm for the compensation mode and (6.675 65 ± 0.000 45) × 10^−11^ m^3^ kg^−1 ^s^−2^ with a relative uncertainty of 67 ppm for the deflection mode. The combined value was *G* = (6.675 59 ± 0.000 27) × 10^−11^ m^3^ kg^−1 ^s^−2^ with a relative uncertainty, taking account of correlations, of 40 ppm.

In 2014, the BIPM group rebuilt and replaced most of the apparatus. Lots of key parameters were improved, including the test and source mass coordinates, calibration of the angle measurements, and precision of the torque measurements and timing measurements. The compensation and deflection modes yielded respective values of *G* of (6.675 15 ± 0.000 41) × 10^−11^ m^3^ kg^−1 ^s^−2^ and (6.675 86 ± 0.000 36) × 10^−11^ m^3^ kg^−1 ^s^−2^ with relative uncertainties of 61 ppm and 54 ppm, respectively. The weighted mean was (6.675 54 ± 0.000 16) × 10^−11^ m^3^ kg^−1 ^s^−2^ with a relative uncertainty of 24 ppm, taking account of all correlations. The new value is just 7 ppm below the BIPM-01 result. However, the BIPM-01 and BIPM-14 values are larger than the CODATA 2014 recommended value by 226 ppm and 219 ppm, respectively.

### UWup-02

Compared with the torsion balance, the Fabry–Pérot pendulum consists of two simple pendulums used as test masses. By placing the source masses on the axis defined by the line joining the test masses, the distance between two simple pendulums is changed when the source masses are placed at different sites. The change in the resonance frequency is measured to calculate the gravitational constant. By using the high sensitivity of the resonance frequency measurement of the Fabry–Pérot cavity, the change of position between two simple pendulums caused by gravitational interaction can be precisely measured. The measurement accuracy of *G* is directly dominated by that of the length of the cavity. Meanwhile, the Fabry–Pérot cavity composed of two simple pendulums can reduce the influence of ground tilt motion effectively through the differential mode.

The Fabry–Pérot pendulum was used to measure *G* at the University of Wuppertal, Germany, by Meyer *et al.* [24,65,66]. The final result was reported by Kleinevoß in his doctoral thesis in 2002 [33]. The schematic of the experiment is given in Fig. 3(c). The heart of the apparatus was a microwave Fabry–Pérot resonator, the two reflectors of which used as test masses were independently suspended by tungsten fibres. Two source masses, placed on each outer side of the resonator, moved symmetrically and simultaneously from a reference position to a measuring position. This process caused the distance between two test masses of the cavity to change, which in turn changed the resonant frequency of the resonator. As a result, the gravitational constant was determined with a relative uncertainty of 150 ppm to be (6.674 22 ± 0.000 98) × 10^−11^ m^3^ kg^−1 ^s^−2^.

### MSL-03

Armstrong and Fitzgerald, at the Measurement Standard Laboratory of New Zealand, published the value of *G* in 2003. Like the BIPM experiment, they also used the electrostatic compensation method but with totally different apparatus. The schematic of the MSL-03 experiment is given in Fig. 3(d). Two large cylindrical masses were adopted as the source masses to produce the gravitational interaction on the small copper cylindrical test mass that was suspended from a tungsten fibre instead of a torsion strip. When the test mass rotated due to the source masses attraction, the signal turned on the feedback control to generate a voltage applied to an electrometer. Then the electrostatic force on the test mass compensated for the gravitational force so that the fibre did not experience any deflection. The value of *G* could be determined by the voltage. In the MSL experiment, the electrostatic torque constant was determined in a separate experiment by measuring the angular acceleration of the test mass when the source masses were removed and a voltage was applied to the electrometer.

The MSL group performed precise measurements of *G* using the MSL torsion balance for more than ten years [23,34,67–69]. Over that time, the experimental devices, the measurement method and the data analysis process have been improved. In 2003 they combined four results, three of which used copper source masses and one used stainless steel source masses, and obtained the final value of (6.673 87 ± 0.000 27) × 10^−11^ m^3^ kg^−1 ^s^−2^ with a relative uncertainty of 40 ppm [34]. However, this value is not consistent with the values of BIPM-01 and BIPM-14 in the compensation mode by 249 ppm and 192 ppm, respectively.

### HUST-05, HUST-09 and HUST-18

Our group from Huazhong University of Science and Technology (HUST) began determining the value of the gravitational constant in the 1980s. The first value of *G*, named HUST-99, was obtained and published in 1998 [30] using the time-of-swing method. Then two systematic errors were found and the value of *G* was corrected and reported in 2005, named HUST-05 [35]. In the following experiment using the same method, new apparatus was designed and built, with several improvements made to greatly reduce the uncertainties. Finally, the result was reported in 2009, named HUST-09 [37,38]. Taking into consideration the large discrepancy among values of *G* that may be caused by undiscovered systematic errors, our group performed a new measurement with two independent methods, the ToS method and the AAF method, in the same laboratory. The two values of *G* obtained using the different methods were published in 2018, named HUST-18 [50].

The time-of-swing method has a long history in the measurement of the gravitational constant and has been widely used [19–21,25,26,30,35,37,38,44]. The basic principle is to measure the change of the pendulum period when the source mass is placed at different positions, named ‘near’ and ‘far’. The main advantage of the ToS method is that it converts a small change in the weak gravitational force into an oscillation frequency, so that a more accurate value of *G* can be achieved. However, the main disadvantage is that the measurement results are severely dependent on the constancy of the spring constant of the torsion fibre.

In early experiments measuring *G* using the ToS method [19–21], the spring constant *k* of the torsion fibre was considered to be constant. However, research examining the internal friction of the fibre suggested that there existed a frequency-dependent property of the spring constant, namely the anelasticity effect. In 1995, based on the assumption of a continuum Maxwell model, Kuroda from the University of Tokyo proposed that the anelasticity of the fibre could produce an upward fractional bias of 1/(π*Q*) in the *G* measurement using the ToS method [64], where *Q* is the quality factor of the main torsion mode (e.g. in general, tungsten fibre with a *Q* value of about 2000 will introduce an anelasticity effect of about 160 ppm to the *G* value). In 2000, Newman and Bantel from the University of California at Irvine further enlarged Kuroda’s 1/(π*Q*) hypothesis to the range between 0 and 1/(2*Q*) according to the general continuum Maxwell model [70,71]. Nevertheless, this conclusion gives only a range for the anelasticity effect, which cannot yield an exact correction for the *G* value. To measure the anelasticity effect, Kuroda and his colleagues designed an experiment to examine the relationship between the spring constant of tungsten fibre and the oscillation frequency of the torsion pendulum by changing the moment of inertia of the dumbbell pendulum [72]. Unfortunately, they did not obtain a definite result to confirm his hypothesis due to a relatively large measurement error in the moment of inertia. This conjecture was subsequently proved by Bagley and Luther from Los Alamos National Laboratory indirectly [26]. They performed *G* measurements with the ToS method using an uncoated tungsten fibre with a *Q* value of 950 and a gold-coated fibre with a *Q* value of only 490. According to Kuroda’s 1/(π*Q*) hypothesis, the anelasticity effect introduced about 300 ppm to the *G* value. By applying a correction, the two values of *G* agreed quite well with each other, within the 1σ range. However, the relative standard uncertainties of the two values were 165 ppm and 122 ppm, respectively, which means that their experiment was not sufficient to confirm this hypothesis precisely.

**Figure 4.**
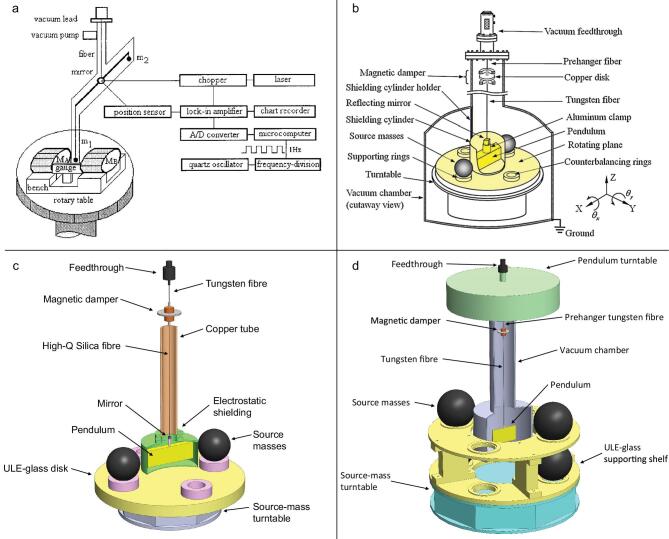
(a) Schematic of the HUST-99 and HUST-05 apparatuses [30]. Copyright 1998, The American Physical Society. (b) Schematic of the HUST-09 apparatus [37]. Copyright 2009, The American Physical Society. (c) Schematic of the HUST-18 apparatus using the time-of-swing method. (d) Schematic of the HUST-18 apparatus using the angular acceleration feedback method.

In the HUST-99 experiment (see Fig. 4(a)), two separated nonmagnetic stainless steel cylindrical source masses, which were placed outside the vacuum chamber, were located on opposite sides of the copper spherical mass 1 (*m*_1_), which hung from one end of the beam. The counterweight mass 2 (*m*_2_) was fixed on the other end of the beam. The torsion pendulum was suspended by a tungsten fibre inside the vacuum chamber. The gravitational signal was obtained by measuring the changes in the period of the pendulum with and without the source masses. In 1998, the *G* value was measured to be (6.6699 ± 0.0007) × 10^−11^ m^3^ kg^−1 ^s^−2^ with a relative uncertainty of 105 ppm [30]. In the following seven years, two systematic errors, the eccentricities of the mass center from the geometric center of cylindrical source masses and the air buoyancy effect, were discovered. Taking these two corrections into account, the value of *G*, named HUST-05, was (6.672 28 ± 0.000 87) × 10^−11^ m^3^ kg^−1 ^s^−2^ with a relative uncertainty of 130 ppm [35,73]. In 2016, the CODATA Task Group reevaluated the anelasticity that existed in the HUST-05 experiment. Since the *Q* factor of the torsion balance was approximately 3.6 × 10^4^, the bias due to fibre anelasticity was originally neglected. For the CODATA 2014 adjustment, based on the anelasticity correction, the HUST-05 value has been reduced by 8.8 ppm to a value of (6.672 22 ± 0.000 87) × 10^−11^ m^3^ kg^−1 ^s^−2^ [4].

In order to overcome the difficulties encountered in the HUST-99 experiment, we presented a new value of the gravitational constant *G* using the ToS method, as shown in Fig. 4(b), named HUST-09. Four major improvements were adopted to greatly reduce the uncertainties. The first improvement was to directly measure the anelasticity effect of the fibre. During the HUST-09 experiment, the anelasticity introduced about 200 ppm, as predicted by

Kuroda [64], and Newman and Bantel [70,71] due to an annealed and thoriated tungsten fibre with a *Q* factor of about 1700. In a separate experiment with two additional disk pendulums this bias was determined to be -211.80(18.69) ppm [74]. The second improvement was to use spherical source masses instead of cylinders, which minimized the effects of density inhomogeneity and eccentricities. The third improvement was to use a quartz block pendulum, which simplified its vibration modes and minimized the uncertainty of the moment of inertia. The fourth improvement was to set both the pendulum and source masses in a vacuum chamber, which reduced the error of measuring the relative positions. Finally, the combined value of *G* in the HUST-09 experiment was (6.673 49 ± 0.000 18) × 10^−11^ m^3^ kg^−1 ^s^−2^ with a relative uncertainty of 27 ppm [37].

The HUST-09 value was the most precise value that was determined with the ToS method at that time. However, there are still some large systematic errors, especially the anelasticity effect. Therefore, an improved experiment with high accuracy and high confidence level needed to be carried out. In the HUST-18 experiment, we also adopt the ToS method, based on our extensive experience in this field [73–83], but we use a high-*Q* silica fibre instead of a tungsten fibre in order to reduce the anelasticity effect. Furthermore, other large systematic uncertainties encountered in the previous experiments are also minimized, including the coating layer, clamp, ferrule. Meanwhile, we also chose the AAF method due to its advantage of being independent of anelasticity.

The schematic of the HUST-18 experiment using the ToS method is given in Fig. 4(c). The apparatus was very similar to that used in the HUST-09 experiment. The pendulum, which was an aluminum-coated fused silica block, was suspended by a thin fused silica fibre. To reduce the potential electrostatic effect, each fibre’s surface was coated with a germanium layer and bismuth layer. The source masses, the turntable and the measurement process were the same as those used in the HUST-09 experiment. During the HUST-18 experiment, two identical apparatuses were used to check for possible errors induced by the setup. For apparatus 1 we used three different high-*Q* silica fibres, and for apparatus 2, which was located about 150 m away in another room, we used a new silica fibre with similar pendulum and source masses. The values of *G* for the four fibres were (6.674 187 ± 0.000 091) × 10^−11^ m^3^ kg^−1 ^s^−2^, (6.674 237 ± 0.000 219) × 10^−11^ m^3^ kg^−1 ^s^−2^, (6.674 269 ± 0.000 093) × 10^−11^ m^3^ kg^−1 ^s^−2^ and (6.674 061 ± 0.000 104) × 10^−11^ m^3^ kg^−1 ^s^−2^ with relative uncertainties of 13.67 ppm, 32.88 ppm, 13.96 ppm and 15.59 ppm, respectively. These four values represented good consistency within the relative uncertainties. Taking into account the correlation between all fibres, the weighted mean of *G* for the ToS method is (6.674 184 ± 0.000 078) × 10^−11^ m^3^ kg^−1 ^s^−2^ with a relative uncertainty of 11.64 ppm [50].

The schematic of the HUST-18 experiment using the AAF method is given in Fig. 4(d). Our group has performed proof-of-principle experiments since 2008 [84,85], redesigning and completely rebuilding the apparatus to reduce several sources of uncertainty that existed in the previous experiment. The pendulum, a gold-coated fused silica block, was suspended from a tungsten fibre in the vacuum chamber supported by an air-bearing turntable. Four stainless-steel spheres, which were located outside of the vacuum chamber, were used as the source masses, supported by ultra-low thermal expansion material shelves with upper and lower layers installed on the coaxial separate turntable. Three different conditions were investigated with the same apparatus. The signal frequency of interest in condition 1 was different from that in the other two conditions. In condition 3, the different group members performed two additional improvements and repeated the whole experimental process independently. The three conditions yielded values of *G* of (6.674 534 ± 0.000 083) × 10^−11^ m^3^ kg^−1 ^s^−2^, (6.674 375 ± 0.000 082) × 10^−11^ m^3^ kg^−1 ^s^−2^ and (6.674 535 ± 0.000 075) × 10^−11^ m^3^ kg^−1 ^s^−2^ with relative uncertainties of 12.45 ppm, 12.27 ppm and 11.21 ppm, respectively. Taking into account the correlation in all conditions, the weighted mean of *G* for the AAF method is (6.674 484 ± 0.000 078) × 10^−11^ m^3^ kg^−1 ^s^−2^ with a relative uncertainty of 11.61 ppm [50].

The two *G* values obtained in the HUST-18 experiment with different methods have the smallest uncertainties reported to date, and both agree with each other within a 3σ range. Schlamminger from the National Institute of Standards and Technology published the views to emphasize that our study was an example of excellent craftsmanship in precision measurements [49].

### UZur-06

Besides the torsion balance, another instrument that can be used to measure *G* is a beam balance. The principle of determining the *G* value is to measure the change in the equilibrium position of the beam balance when the test mass is affected by the gravitational attraction of the source mass. The beam balance measures the force in the vertical direction. Compared with the torsion balance, the impact of the fluctuation of the background gravity field in the horizontal direction is relatively small on the measurement results, unless the massive objects are located very close to the device. Meanwhile, the larger source mass can be used to increase the gravitational signal. The larger the source mass, the more similar the object is to the point mass. Hence, the effect of the density inhomogeneity of the source mass is smaller than in other methods. The main disadvantage of the beam balance is that the sensitivity is not as high as that of the torsion balance. Because of this disadvantage, its quality factor *Q* does not normally exceed 100, while the torsion balance easily exceeds 1000. The ambient temperature fluctuation will affect the arm length of the beam balance, thus affecting the measurement accuracy, which requires an extremely stringent situation for environmental temperature control. The ground tilt also affects the measurement accuracy.

**Figure 5.**
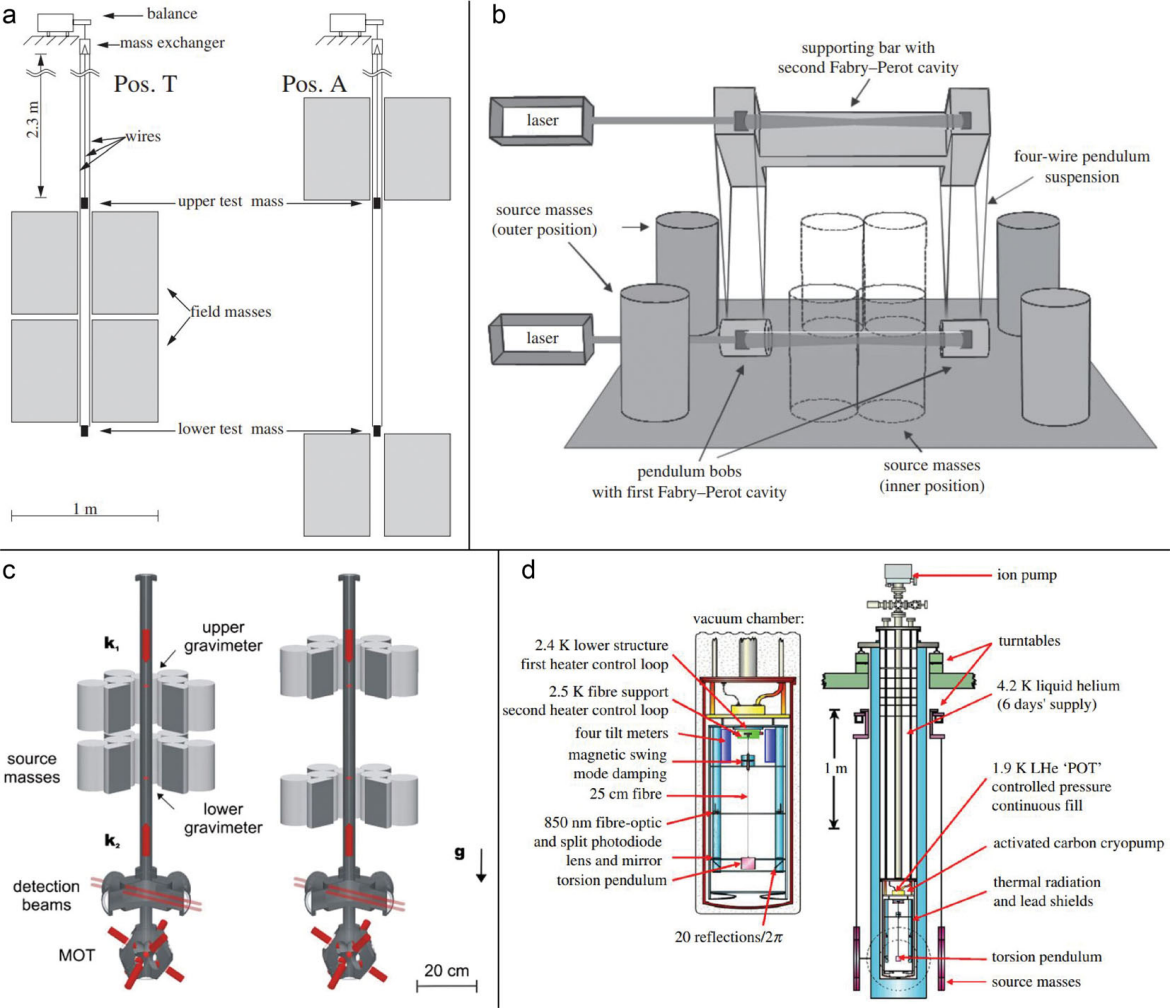
(a) Schematic of the UZur-06 apparatus [36]. Copyright 2006, The American Physical Society. (b) Schematic of the JILA-10 apparatus [94]. Copyright 2014, The Royal Society. (c) Schematic of the LENS-14 apparatus [98]. Copyright 2008, The American Physical Society. (d) Schematic of the UCI-14 apparatus [44]. Copyright 2014, The Royal Society.

An experimental group in the University of Zürich used this approach to measure the *G* value. A schematic of the UZur-06 experiment is given in Fig. 5(a). The device consisted of two test masses and two moveable source masses. The test masses suspended from the long wires were alternately weighed on the beam balance. Their different weights occurred when the source masses were in different positions, labeled ‘Pos. T’ and ‘Pos. A’. The weight difference was the gravitational signal that could determine the *G* value. A preliminary result was reported in 1999 with a relative uncertainty of 220 ppm [86]. In 2002, the gravitational force of two stainless steel tanks filled with mercury instead of water as the source masses on test masses was measured. After carefully analysing the data and the experimental error, they yielded *G* = (6.674 07 ± 0.000 22) × 10^−11^ m^3^ kg^−1 ^s^−2^ with a relative uncertainty of 33 ppm [87]. The Zürich experiment published a final value of *G* in 2006 [36] of *G* = (6.674 25 ± 0.000 12) × 10^−11^ m^3^ kg^−1 ^s^−2^ with a relative uncertainty of 19 ppm. It is worth noting that this value is in good agreement with that of the UWash-00 experiment using a different method. The relevant details of the experiment have been summarized in the final report, two theses [88,89] and several shorter reports [29,36,86,87,90–93].

### JILA-10

In 2010, Parks and Faller published the *G* value using the two simple pendulums method [39,94] similar to that of Kleinevoß’s experiment [33]. In Fig. 5(b) we shows a schematic of the apparatus. A Fabry–Pérot interferometer measured the separation between the two copper test masses that were suspended from four wires with respect to a suspension-point-located reference cavity. Four tungsten cylindrical source masses were supported on air bearings to move to different positions to produce the horizontal gravitational force on each test mass. During the measurement, the source masses were moved between the inner and outer positions several times. By measuring the signal of the difference separation of the test masses, the *G* value could be calculated. Compared to Kleinevoß’s experiment, it is worth noting that there are two major improvements used in the JILA-10 experiment. One improvement is that a laser is used rather than a microwave interferometer. The resolution of the distance sensing is improved due to reducing the wavelength from centimeters to several hundreds of nanometers. The other improvement is the simultaneous measurement of a second Fabry–Pérot cavity, which is attached to the support of the pendulums in order to compensate thermal drifts of the apparatus. Parks and Faller spent six years carefully checking every detail of the experiment, publishing the final result of *G* = (6.672 34 ± 0.000 14) × 10^−11^ m^3^ kg^−1 ^s^−2^ with a relative uncertainty of 21 ppm.

In 2019, authors found two calculation errors related to the rotation of the pendulum bob as the pendulum was displaced. The four wires that suspended the bob were designed to allow the bob to translate without rotation. However, differential loading of the wires caused a small amount of rotation. One of the calculation errors was that they made a mistake in accounting for this rotation when deriving the pendulum spring constants from the period of free oscillation. The correction to the *G* value should be +4.0(0.3) ppm rather than the original value of +58(4) ppm. The other calculation error was that the author did not consider the Abbe error due to the rotation and the fact that the Fabry–Pérot interferometer axis was displaced above the horizontal plane containing bob centers of mass. This correction introduced +94(30) ppm to the value of *G*. The result of correcting these two errors was an increase in the value of *G* by 39 ppm, so the final value should be (6.672 60 ± 0.000 25) × 10^−11^ m^3^ kg^−1 ^s^−2^ instead of (6.672 34 ± 0.000 14) × 10^−11^ m^3^ kg^−1 ^s^−2^, and an increase in the relative uncertainty from 21 ppm to 37 ppm [51]. Although Faller *et al.* and Kleinevoß both used the Fabry–Pérot cavity method, the two *G* values unfortunately differ by 243 ppm.

### LENS-14

Besides the torsion balance, beam balance and simple pendulum, there is another novel technique used in the field of precision measurements of the gravitational constant called cold-atom interferometry. An atomic gravity gradiometer is adopted to determine the differential acceleration experienced by two free-falling samples of laser-cooled atoms under the influence of a nearby source mass. The *G* value can then be calculated through the acceleration and the mass distributions of the source mass and atoms. Tino *et al.* from the University of Florence performed a new determination of the gravitational constant based on the cold-atom interferometry method. In Fig. 5(c) we show a sketch of the experiment. At the bottom of the vacuum chamber, a magneto-optical trap collected rubidium atoms as the test masses. Subsequently, the atoms were launched vertically along the symmetry axis of the vacuum tube. During the launch sequence, the atoms were laser cooled to a temperature of 4 μK. The source masses [95] were tungsten alloy cylinders and could be moved in two different positions near the atom interferometer.

Proof-of-principle experiments to measure *G* using atom interferometry have been reported [96–98]. The Florence group obtained the final result of *G* = (6.671 91 ± 0.000 99) × 10^−11^ m^3^ kg^−1 ^s^−2^ with a relative uncertainty of 150 ppm [42,43]. This novel experiment is exciting because it uses modern tools to solve an old problem [47].

### UCI-14

In 2014, an experimental group at the University of California Irvine performed the experiment of Newton’s gravitational constant with a cryogenic torsion pendulum operating below 4 K using the time-of-swing method. Three high *Q*-value fibres, the as-drawn CuBe fibre, the heat-treated CuBe fibre and the as-drawn Al5056 fibre, were used in order to minimize experimental bias from fibre anelasticity. The *Q* value of each fibre is 82 000, 120 000 and 164 000, respectively. The experimental apparatus is shown in Fig. 5(d). A fused silica plate that was suspended from a long fibre was located in a liquid helium dewar with a temperature control system; hence, the temperature at the suspension point was typically maintained within ± 10 μK during a day or more. Two large copper rings used as the source masses hung outside of the dewar.

The final values of *G* and uncertainties for the three fibres are as follows: *G*_1_ = (6.674 35 ± 0.000 10) × 10^−11^ m^3^ kg^−1 ^s^−2^ with a relative uncertainty of 14 ppm; *G*_2_ = (6.674 08 ± 0.000 15) × 10^−11^ m^3^ kg^−1 ^s^−2^ with a relative uncertainty of 22 ppm; *G*_3_ = (6.674 55 ± 0.000 13) × 10^−11^ m^3^ kg^−1 ^s^−2^ with a relative uncertainty of 20 ppm. The maximum discrepancy of the three values is about 70 ppm; however, there is no explanation for the inconsistency. The authors just speculated that the *G* value determined with the Al5056 fibre may be the least subjected to fibre-associated systematic error because of its higher *Q*. Hence, the unweighted mean of *G* is (6.674 33 ± 0.000 13) × 10^−11^ m^3^ kg^−1 ^s^−2^ with a relative uncertainty of 19 ppm [44]. In 2016, the CODATA Task Group decided to use a weighted mean of the three values and the final UCI-14 adopted in CODATA 2014 is (6.674 35 ± 0.000 13) × 10^−11^ m^3^ kg^−1 ^s^−2^ with a relative uncertainty of 19 ppm.

The UCI group has contributed to this work over 18 years [44,70,99,100]. This experiment has two main advantages. First, the main systematic error from the anelasticity of the fibre is reduced to less than 5 ppm due to the fact that the *Q* factor is increased from a few thousand to over 100 000. Second, the thermal noise and the fibre properties related to temperature variation are greatly reduced because of the extremely low temperature condition.

## CONCLUSION AND OUTLOOK

One of the most basic research fields in physics is to measure the Newtonian gravitational constant precisely and to study the properties of this constant thoroughly. Since Cavendish’s first laboratory measurement of the *G* value using torsion balance over 200 years ago [6], experimenters have devoted tremendous efforts to investigating many possible contributions to the measurement uncertainty, but the relative uncertainty of *G* has not been greatly improved [4,5,14–18]. Specifically, in the past 20 years, many high-precision experiments [21,25,26,31–39,41,42,44,50] have proved that the precise measurement of *G* is an extremely complex and difficult task that requires high-level experimental technology and methods. The measurement of *G* is not only of great significance to the understanding of the gravitational interaction, but also to geophysics, astrophysics and cosmology. Meanwhile, it can promote the development of precision measurement technology.

Measuring *G* accurately is challenging. The reduction in the uncertainty of *G* has been improved by only two orders of magnitude over more than two centuries. The difficulty is that the gravitational interaction is weak and cannot be screened. In addition, *G* has no known, confirmed dependence on any other fundamental constant. With the development of science and technology in recent years, experimenters have performed some novel techniques to improve the sensitivity of experiments. Several new methods, such as cryogenic torsion balance and cold-atom interferometry, have been adopted for the *G* measurement and lots of precise results have been obtained. Unfortunately, there is still a large discrepancy of about 550 ppm among the thirteen values of *G* reviewed in this paper, even though the relative standard uncertainties of many results have been less than 50 ppm.

Why is the scatter of the *G* values so large? In principle, there are two possibilities in science and technology that can explain the obvious inconsistency. The first is that there could be systematic errors that are not fully understood in some or all the experiments. Usually, experimenters take great care to investigate the systematic errors and apply the correction to the result in order to confirm that it is the best estimate of the true value. A bias, however, may be present in the published value that the experimenters do not realize. For example, Faller and Parks obtained their data in 2004, but have spent six years searching and estimating the systematic errors that might have been missed. In 2019 they found two calculation errors [51], which increased the *G* value by 39 ppm compared with the published value in 2010 [39]. A similar situation, but lasting 65 years, began with Heyl, who measured the gravitational constant with torsion balance using the ToS method in 1930 [19]. After that, the ToS method became the most widely used method for the *G* measurement. In 1995, Kuroda proposed that the anelasticity of the fibre leads to an upward bias of 1/(π*Q*) in the *G* value with this method [64]. This systematic error can introduce 100–300 ppm to the *G* value using the metal fibre. The second possibility is that there might be some unknown physical mechanism to explain the discrepancy in the *G* values. However, compared with the first possibility, it is a relatively long-term task to confirm a new physics that requires accumulating extensive experimental data to find the nature of the interaction.

Besides the scientific and technical reasons discussed above, there might be two additional facts that exist in the measurement of *G* among most of the experimental groups. One is that most *G* measurement groups have only a few experimenters. The other is that no groups repeated the experiments using the same devices and method after publishing the results. For the published values discussed above, nine experimental groups and seven different methods were involved. Based on the author list of each publication, there were no more than five experimenters in each of the seven groups, and only two of the seven groups continue to perform the *G* measurement. Meanwhile, according to the papers published by each group, no two identical *G* experiments have ever been repeated. It should be noted that repeating independently is very significant for the scientific research.

In July 2019, the conference titled the ‘22nd International Conference on General Relativity and Gravitation & 13th Edoardo Amaldi Conference on Gravitational Waves’ was held in Valencia, Spain. One of the parallel sessions in this conference, called ‘Measurements of *G*’, chaired by Schlamminger aimed to resolve the problem of discrepancy among recent *G* values and discover new methods for measuring the gravitational constant. A working group composed of many *G* measurement scientists was established during this parallel session for the primary purpose of supporting experimental efforts to measure *G*. The working group suggested holding regular meetings to discuss future experiments, proposals and new ideas, and to also consider the possible mechanisms to explain or evaluate the existing discrepancies between each experimental result.

**Figure 6.**
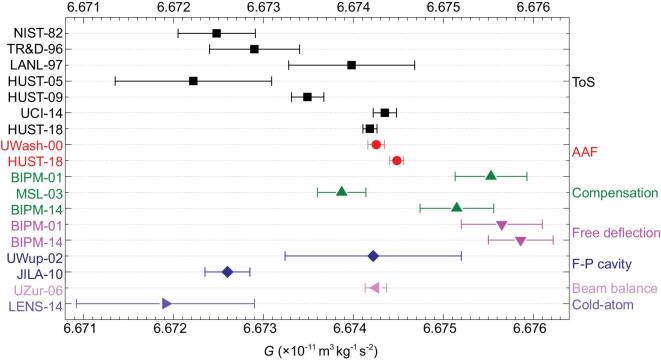
The *G* values adopted in the CODATA 2014 recommended value and two values of HUST-18. The labels on the left denote the identification of each value, and the labels on the right denote the different methods. The same symbol and color represent use of the same method.

For the future development of the *G* measurement, the main target should be to reduce the discrepancy of every value of *G*. In Fig. 6 we show the *G* values adopted in the CODATA 2014 recommended value and two values of HUST-18, where two results obtained by Quinn *et al.*, named BIPM-01 and BIPM-14, represent two individual *G* values determined using different methods. Therefore, 18 values are divided into seven methods and marked with different colors. It is clear that even when using the same method, there is an obvious inconsistency between the obtained results. So every group needs to repeat their experiments using the same method and devices, and should make much more effort to estimate the potential systematic errors. The first step is to confirm that the values obtained with the same method are consistent with each other within a 1σ range. After that, different groups should strengthen the international cooperation to discuss the possible undiscovered systematic errors among different methods. In general, it is hoped that more and more scientists could be involved in the *G* measurement and the problem of ‘Big *G*’ can be solved in the near future.
